# The long-distance relationship between *Dirofilaria* and the UK: case report and literature review

**DOI:** 10.3389/fvets.2023.1128188

**Published:** 2023-04-27

**Authors:** Rossella Panarese, Rhiannon Moore, Antony P. Page, Mike McDonald, Emma MacDonald, William Weir

**Affiliations:** ^1^School of Biodiversity, One Health and Veterinary Medicine, College of Medical, Veterinary and Life Sciences, University of Glasgow, Glasgow, United Kingdom; ^2^Dundas Veterinary Group Limited, Edinburgh, United Kingdom

**Keywords:** *Dirofilaria repens*, *Dirofilaria immitis*, heartworm disease, subcutaneous dirofilariosis, mosquito-borne disease, dirofilariasis, United Kingdom

## Abstract

Over the last two decades, vector-borne pathogens (VBPs) have changed their distribution across the globe as a consequence of a variety of environmental, socioeconomic and geopolitical factors. *Dirofilaria immitis* and *Dirofilaria repens* are perfect exemplars of European VBPs of One Health concern that have undergone profound changes in their distribution, with new hotspots of infection appearing in previously non-endemic countries. Some areas, such as the United Kingdom, are still considered non-endemic. However, a combination of climate change and the potential spread of invasive mosquito species may change this scenario, exposing the country to the risk of outbreaks of filarial infections. Only a limited number of non-autochthonous cases have been recorded in the United Kingdom to date. These infections remain a diagnostic challenge for clinicians unfamiliar with these “exotic” parasites, which in turn complicates the approach to treatment and management. Therefore, this review aims to (i) describe the first case of *D. repens* infection in a dog currently resident in Scotland, (ii) summarise the available literature on *Dirofilaria* spp. infections in both humans and animals in the United Kingdom and (iii) assess the suitability of the United Kingdom for the establishment of these new VBPs.

## 1. Introduction

The zoonotic mosquito-borne filarial nematodes *Dirofilaria immitis* (Leidy, 1856) and *Dirofilaria repens*, Railliet and Henry, 1911, are among the most important canine vector-borne pathogens (VBPs) of One Health concern in mainland Europe (1, 2). Both *Dirofilaria* species are widely distributed in tropical and temperate regions (3, 4) and can infect a wide range of animals and humans. *Dirofilaria immitis* is the causative agent of canine heartworm disease (HWD), a severe syndrome that can be fatal if not promptly diagnosed and properly treated. The closely related parasite, *D. repens*, is the causative agent of subcutaneous dirofilariosis (SCD), which is generally asymptomatic or paucisymptomatic, and is often only reported as an incidental finding during unrelated surgical or clinical procedures (3). Although humans are not the definitive host of these mosquito-borne filaroids, they may still develop symptoms. In fact, *D. repens* causes subcutaneous and/or ocular nodules while *D. immitis* infection results in the development of characteristic ‘coin’ lesions in the lung parenchyma (3). In some cases, *D. immitis* human infections may be misdiagnosed as cancer or pulmonary disease and thus infected individuals may undergo unnecessary and painful surgical procedures and/or be prescribed inappropriate treatment before receiving the correct diagnosis (5, 6). Despite humans occasionally experiencing microfilaraemia due to *D. repens* infection (7), dogs remain the main reservoir of both infections for humans and animals and play a central role in disease epidemiology, especially when competent mosquito vectors and other susceptible hosts coexist in the same environment (8). Almost 70 mosquito species belonging to the genera *Aedes/Ochlerotatus*, *Anopheles* and *Culex* are known to be susceptible to these filarial worms. However, only a proportion of these are considered competent vectors and only a handful of species, including *Aedes albopictus* and *Culex pipiens*, are understood to play a major role in disease transmission (9, 10). Mosquitoes become infected during the blood meal, after ingesting the microfilariae (mfs) released by the adult female in the bloodstream of the mammalian host. Once inside the Malpighian tubules of the invertebrate host, the mfs moult several times before developing into the infective L3 stage larvae which migrate to the mosquito’s *labium*. In this location, they are ready to actively penetrate the skin of the next host during the mosquito’s blood meal (3). The entire process requires from 8 to 21 days depending on ambient temperature and filarial species involved (10, 11). Filarial nematodes are considered to be a re-emerging threat for both humans and animals in the majority of European countries due to an increasing number of cases and the extension of their geographical range (4, 12). Certain areas, such as the United Kingdom, are not considered to be endemic and thus it may be argued that they are exposed to the risk of unexpected outbreaks of infection, as has been documented in other previously non-endemic countries (2, 13).

### 1.1. *Dirofilaria* spp. diagnosis as a distribution and prevalence study-bias

The prevalence of *Dirofilaria* spp. infection in humans and animals is often underestimated and clinical cases can be misdiagnosed, particularly in non-endemic countries (14, 15). This may be explained, in part, by the lack of a single definitive diagnostic method for the efficient, simultaneous detection of both filarial species. This is compounded by the fact that infected individuals may be either asymptomatic or paucisymptomatic, in particular for *D. repens* in dogs and *D. immitis* in humans, and that symptoms/clinical signs, when present, may overlap with other illnesses. This may be further exacerbated by a knowledge gap, on the part of clinicians, on the ever-changing geographical distribution of both *Dirofilaria* species and thus filarial infection may not even be considered as a differential diagnosis in areas where the parasite is usually absent (13, 15, 16). In endemic countries or in geographical areas where at least a low/moderate prevalence has been recorded and consequently there is appreciable awareness in the veterinary community, effective treatment and control measures can result in an observable decrease in incidence of both diseases (4, 12). Several methods have been developed to diagnose dirofilariosis and are currently in use. These may be divided into parasitological techniques for mfs detection in the host’s blood (e.g., fresh blood smear, buffy coat examination, filtration or modified Knott’s test), serological (i.e., enzyme-linked immunosorbent assays (ELISA), rapid immunochromatographic tests (ICT) and molecular approaches (i.e., probe-based quantitative polymerase chain reaction (qPCR), conventional PCR (cPCR), duplex qPCR for the simultaneous detection of both *Dirofilaria* spp.)), together with fine-needle aspirate (FNA) and histological examination of nodules when present (3, 12, 17, 18). Serological tests are available as reference laboratory assays and also point-of care (POC) tests (3, 19–21). While for *D. immitis*, one can rely on rapid and straightforward in-clinic test kits together with several serological laboratory tools, no similar tests are available for *D. repens* (12). Encouragingly, epitopes of *D. repens* have recently been identified by Pękacz and colleagues (22), using phage display technology in combination with a 12-mer peptide library. These antigenic peptides have been shown to be strongly recognised by IgG from sera of infected dogs (22) and these may form the basis of a new generation of serological tests for *Dirofilaria* spp.

According to several studies, antigen tests may be effective in determining infection status earlier than the parasitological concentration assays; the former may be accurate as early as 5 months post infection (p.i.), while the latter require testing at least 7 months p.i. (23, 24). The sensitivity of these tests when used in the field may not be as impeccable as the product sheets imply (23), as it has been recognised since the 1980s that the presence of immune complexes may negatively impact antigen test reliability (23). Consequently, it has been determined that test sensitivity may be improved by subjecting samples to immune complex dissociation (ICD) prior to testing (25, 26). Indeed, ICD has been shown to improve antigen detection in both experimental and natural *D. immitis* infections by releasing HW antigens that are bound to host antibodies (27–29). ICD methods are based broadly on two alternate technical approaches, namely heat-and acid-treatment. Although heat-treatment is the one most commonly employed (29), acid-treatment with trichloroacetic acid (TCA) has shown similar efficacy without decreasing specificity, a recognised drawback of heat-treatment (23, 29–32). According to the American Heartworm Society (AHS) and the European Society of Dirofilariosis and Angiostrongylosis (ESDA), the diagnosis of *D. immitis* should be demonstrated by the presence of circulating mfs and/or adult antigens (24, 33). However, each diagnostic method has different sensitivity and specificity, and each of them has its own advantages and disadvantages which may lead to false positive or negative results (22–25, 31–33). Indeed, studies on the incidence and/or prevalence of *Dirofilaria* spp. infection in the same canine population have shown differing results according to the diagnostic method adopted (15, 18, 19, 30). For this reason, diagnosis of animal dirofilariosis must rely on the integration of results from at least two methods in order to reduce the risk of false positives and negatives, as recommended by the AHS (24). In human medicine, the diagnostic approach places particular emphasis on clinical examination, anamnesis and travel history of the patient, while additional laboratory methods may include morphological identification of the worm extracted from the nodules, direct detection of DNA of the parasite or of its endosymbiont *Wolbachia* and on other serological tests for detecting antibodies against the filarial antigens (7, 34). In addition to this, modified Knott’s method can be applied also in humans, but it is only helpful in *D. repens* cases when microfilaraemia is present (7). Unfortunately, the combination of a non-specific clinical presentation, the potential delay in seeking medical assistance and frequent initial misdiagnosis, conspires to hamper disease diagnosis and management and thus dirofilariosis is currently considered to be an emerging zoonosis in Europe (34).

### 1.2. *Dirofilaria* spp. in Europe: the latest trend

The epidemiology of vector-borne pathogens (VBPs) has dramatically changed across the globe over the last two decades as a consequence of a variety of environmental, socioeconomic and geopolitical factors (35–39). These changes are, to a large extent, associated with alterations in the distribution and biology of their arthropod vectors. Taken together, this reshaping of VBPs and vector epidemiology has far-reaching implications for both animal and human health (40, 41). *Dirofilaria immitis* and *D. repens* are perfect exemplars of European VBPs that have undergone profound changes in their distribution which in turn have precipitated striking changes in disease epidemiology (1, 2). Several studies have investigated the influence of a global temperature rise on the development time of *Dirofilaria* spp. in the mosquito vector and the impact of this on human and animal health (11, 42). In particular, it has been estimated that the threshold temperature for the extrinsic incubation of the filarial larval stages within the arthropod-vector is 14°C, below which larval development stops (11). Climate change, therefore, represents a clear and present threat which may both directly and indirectly affect the distribution, persistence and spread of these filarial species, exposing new naïve animal and human populations to the risk of infection (2, 13, 42). According to the Intergovernmental Panel on Climate Change (IPCC), there has been a documented recent rise in world temperature of 1.1°C in recent years (43). This has already affected VBP transmission and the further predicted increase in global temperature will only serve to worsen the situation (11). Indeed, all projected distribution studies on *Dirofilaria* spp. employ a climate-based approach to mathematically model the capacity of different geographical areas to support the extrinsic development of the parasite. A geographical area may be classed as suitable for extrinsic development if the following criteria are met: a minimum total environmental heat of 1°C in excess of the threshold of 14°C, for a total of 130 Heartworm Development Units (HDU), evaluated over a mosquito life span of a maximum of 30 days (a longer period being incompatible with mosquito survival) (11, 44). Along with a rise in global temperatures which affects the arthropod vectors, the change in distribution has been ascribed also to an increased movement of animals among non-and endemic countries and to the lack of chemoprophylaxis treatments in non-endemic areas (2). All these factors have increased the numbers of new clinical cases in geographical areas previously described as non-endemic, which has led to the establishment of new hotspot of infections, such as those in southern Europe. For example, in southern Italy, prevalences between 56 and 78% have been recorded, the highest in the Mediterranean regions (2, 13). *Dirofilaria repens*, known to be endemic in the temperate regions, is now extending its range across Central, Eastern and Northern Europe (45, 46). While *D. immitis* is endemic in Southern and Western Europe, it is extending its range into the cooler regions of Central, Eastern and Northern Europe, where it is not yet endemic (4, 46, 47).

## 2. *Dirofilaria* spp. infections in the United Kingdom: the state of the art

The United Kingdom has always been considered a non-endemic country for the presence of *Dirofilaria* spp. (12, 16, 48) and the limited number of human and canine cases of dirofilariosis reported in the United Kingdom have been contracted while in endemic geographical areas such as Italy, Greece, Romania, Spain and Sri Lanka (12, 16, 48, 49). The few case reports recorded only *D. immitis* infection, with no mention of *D. repens* infection in the United Kingdom (3, 16, 50). However, in the United Kingdom, as in the rest of Europe, environmental factors such as climate change, which may facilitate the introduction of new invasive mosquito vectors, and an increased movement of animals and humans across the national borders have affected the parasite incidence dynamic, with an increased number of HW reports over the last two decades (11, 51). Several authors have argued that the progressive relaxation of the Pet Travel Scheme 2000 (PETS) in recent years (i.e., since 2012) is one of the major factors responsible for the spread of VBPs (45, 52). In particular, in the United Kingdom, this relaxation has been accompanied by a huge influx of dogs from abroad (i.e., 300,000 imported dogs), principally from Romania and Spain (12, 16, 53). Nonetheless, the United Kingdom has not been a member of the PETS since the first of January 2021, and it is currently considered a ‘Part II listed non-EU country’. Thus, pets travelling from or to the United Kingdom may be subject to differing health requirements compared to EU countries (54, 55). During the first epidemiological survey conducted in 2010, the filarial-specific antibody prevalence was recorded as 0% over a total of 1,028 dogs tested across the United Kingdom (56). A later European study performed between 2016 and 2020 revealed a canine HW seroprevalence of 5–7% in central/southern England, with no seropositive dogs detected in other parts of the country (57). While the overall seroprevalence of HW infection remains very low (56, 57), there have been two reports of clinical *D. repens* infection in United Kingdom dogs in recent years in the midlands of England (12, 58, 59). Both studies reported infection in imported dogs, the first originating from Romania (59, 60) and the second from Corfu (58). The first report documents a dog living in the United Kingdom for several months before, in 2014, a diagnosis of filarial infection being made, and treatment being instigated. The diagnosis was based on molecular and serological analyses although it was not stated whether mfs were present in the peripheral blood (59). The second report recounts a dog which had recently been relocated to the United Kingdom and in which a 17 cm female nematode was discovered as an incidental finding during routine castration. Again, no information was reported on the presence of mfs (58). Similarly, to the best of our knowledge, only a very limited number of human cases of *Dirofilaria* spp. infection have been recorded in the United Kingdom and these have been attributed to people contracting the disease while living abroad in endemic countries before returning to the United Kingdom (12). For example, a case of subcutaneous dirofilariasis in a 32-year old man was reported by Ahmed and colleagues in 2010 (49). The patient presented with a parotid duct obstruction and a plum-sized swelling on the same side, anterior to his masseter muscle. Initially misdiagnosed as a tumour of the accessory parotid gland, histological examination revealed the presence of an adult *Dirofilaria* spp. nematode and it was established that the man had a history of travelling in Sri Lanka (1, 49). More recently, subcutaneous dirofilariasis was detected in a 67-year-old English man (48). This individual presented with a six-month history of a small painless lump developing on the right side of his abdomen which initially appeared 2 years after a fortnight’s travel in Tuscany, Italy (48). Currently, the United Kingdom is still considered a non-endemic country, however in this age of frequent foreign travel, an awareness of clinical dirofilariasis is required among veterinarians and medical practitioners alike.

## 3. Case report in Scotland

**Figure 1:**
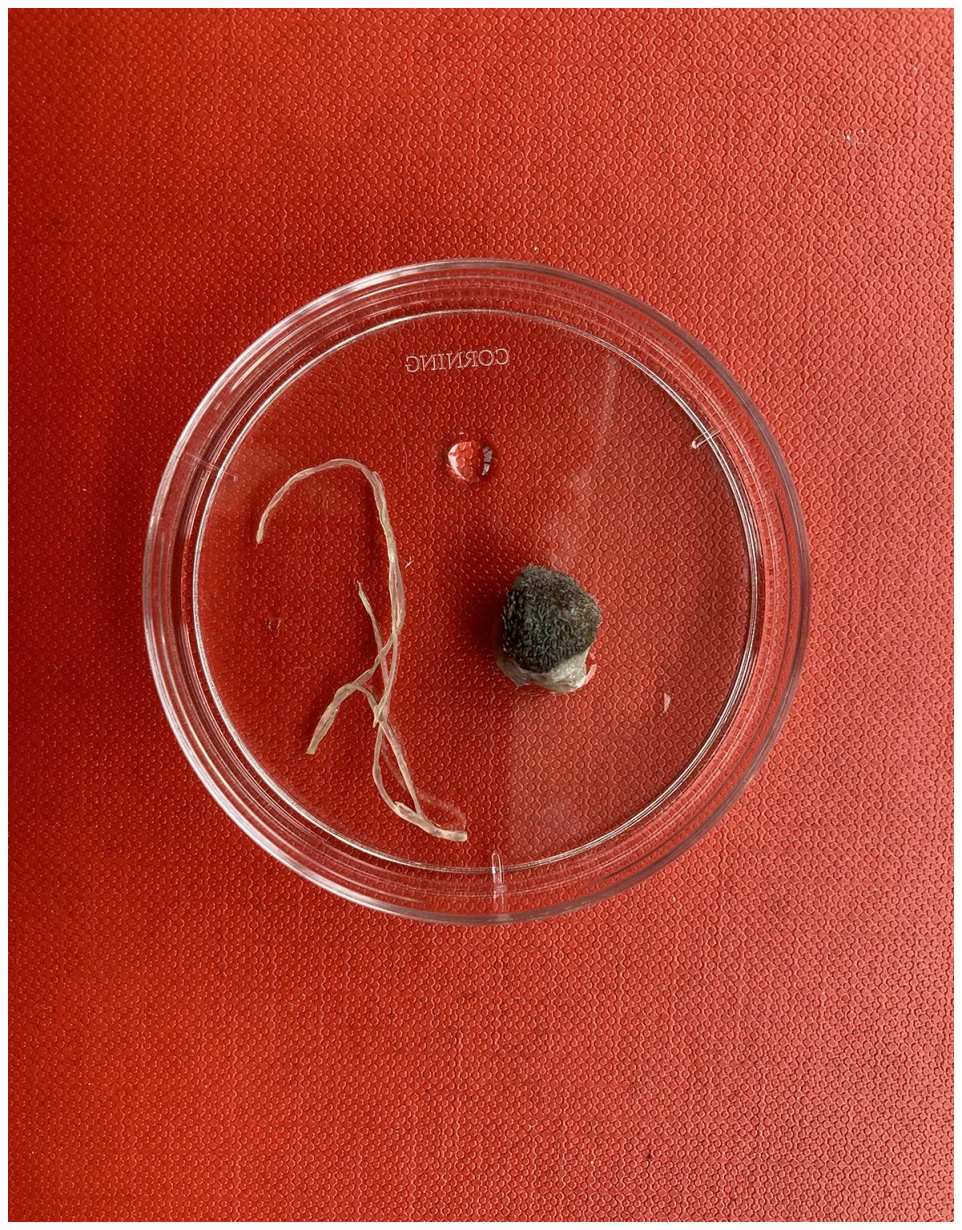
*Dirofilaria repens* adult extracted from the dog’s nodule in a 60 mm Petri dish.

On the 24th of August 2020, a 4-year-old female neutered crossbreed dog was presented at a veterinary clinic in Edinburgh, Scotland with a small, raised lesion on its nasal dorsum (Figure 1). The dog had arrived in the United Kingdom on the 7th of July 2020, having been born in Romania, where it had been kept in a dog shelter since it was a puppy. Apart from treatment for *Echinococcus multilocularis*, which is necessary for entry into the United Kingdom (i.e., praziquantel, (61)), the dog arrived with no additional record of endo-or ectoparasitic treatments. At first examination, the dog was found to be generally in good health. The nodule was initially described as being a small and firm fluctuating mass on the left side of the dog’s face, above the premolar teeth of the maxillary arch, 206 and 207, and did not appear to be causing any pain. It was recommended that the owner monitor the nodule over the time and report any change in its dimensions.

About 1 month after the first clinical examination, the dog was treated with Milbemax® (12.5 mg milbemycin oxime and 125 mg praziquantel, Elanco United Kingdom AH Limited) and Bravecto® (500 mg fluralaner, MSD Animal Health S.r.l.), a standard prophylactic protocol for endo-and ectoparasites. It subsequently received the same anti-parasitic treatment every three-months. Over the following 2 years, the dog was seen by the practice for problems related to anxiety and weight loss (1 kg over 4 months) with the latter being mostly attributed to the dog being a fussy eater. Subsequent biochemical analyses of the dog’s blood revealed no anomalies and therefore the owner decided against further investigation.

On the 25th of July 2022, the dog was again presented to the clinic as the owner had become concerned about the nodule on the dog’s nose. This had grown since the initial consultation and was now approximately 2 cm in diameter and, thus, a FNA was performed under sedation. It was observed that the material inside the nodule was mucopurulent in nature and the dog was treated with a broad spectrum antibiotic (250 mg Clavaseptin®) and an anti-histamine (4 mg chlorphenamine) for 1 week. Cytological analysis of the FNA confirmed a severe neutrophilic inflammation, but no microorganisms or neoplastic cells were observed. Still considering the remote possibility of a neoplastic origin of the nodule or the presence of a fistulous tract originating from a tooth root abscess, the dog underwent another week of treatment. Following radiography, which produced no evidence of a dental fistula, the mass was removed and found to be a series of cysts which were well-organised around a nematode-like body. The structure, tentatively suspected of being an aberrantly located *Thelazia callipaeda*, was stored in a 70% ethanol solution and referred to the Veterinary Diagnostic Service (VDS) at the University of Glasgow School of Veterinary Medicine, for parasitological and molecular analyses together with a 2 ml sample of blood in sodium citrate.

**Figure 2:**
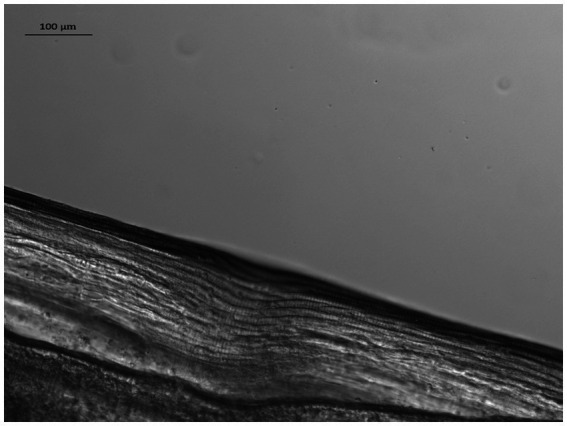
Longitudinal cuticular ridges of *Dirofilaria repens* adult in detail.

At the VDS laboratory, following suspension in 10% glycerol solution, the nematode’s length was measured and its sex determined using light microscopy. Measurements and photographs were taken using a Zeiss Axioskop 2 microscope, with Zeiss Mrm camera and Axioscope software, while the identification was carried out using previously published morphological keys (62, 63). The nematode had a whitish color, narrow rounded ends and a total length of 13.0 mm. It was highly dehydrated with the anterior extremity showing evidence of decomposition and crushing. It was therefore difficult to accurately measure the width of the anterior end and its distance from the nerve ring and the vulva. However, characteristic fine longitudinal cuticular ridges (Figure 2) visible along the whole body allowed its identification as *D. repens*. In addition, although no mfs were present in the uterus, it was identified as a female specimen due to the absence of the *spicula* and the length of its body.

The dog’s blood sample was processed using a modified Knott’s test for the detection of potentially circulating mfs (64). In order to confirm the nematode’s identity and to exclude the occurrence of other filarial infections, it was analysed by polymerase chain reaction (PCR). Genomic DNA was extracted from a portion of its body and from 200 μl of whole blood using a Blood & Tissue® nucleic acid extraction kit (Qiagen, Germany) and screened by conventional PCR using generic primers targeting the 12S rDNA and *COX1* locus of filarial nematodes (17, 65, 66). The amplicons were gel purified using a QIAquick® Gel extraction kit (Qiagen, Germany), then sequenced and compared with the GenBank non-redundant nucleotide database using the Basic Local Alignment Search Tool (BLAST).^1^ The highest ranking ‘hits’ for both amplicons were *D. repens* reference sequences, with 99% identity over 586 nucleotides for *COX1* (MT345575) and 100% identity over 237 nucleotides for the 12S locus (KY828984), thus confirming the morphological identification. Finally, the dog was found to be negative for the presence of mfs using the modified Knott’s test and no filarial nematode DNA was detected in the blood by PCR. The dog made an uneventful recovery from surgery and no further health issues have been reported.

## 4. Discussion

This review reports the current status of dirofilariosis in the United Kingdom, presenting the first non-autochthonous *D. repens* case reported in a dog resident in Scotland, which follows on from two previous cases in the English midlands (58, 59). The dog discussed herein received a definitive diagnosis of dirofilariasis 2 years after its arrival from Romania, where it likely has contracted the infection. If the dog had been microfilaraemic and competent mosquito vectors had been present in the locality, local transmission would have been feasible. Fortunately, mfs were not detected in this case and this was fully anticipated, as the dog had been treated on a three-monthly basis with macrocyclic lactones since its arrival in the United Kingdom (67). Despite this anthelminthic regime being prescribed on a prophylactic basis with a relatively low drug dosage, it would still have acted as a “soft-kill” drug against any *Dirofilaria* species present (68). While the use of milbemycin oxime for “soft-killing” may be recommended in some cases, it is considered the least safe choice among the various macrocyclic lactones employed for treating positive animals (68). In other case scenarios, the lack of a prompt diagnosis for HW disease or a sub-optimal treatment plan may have dramatic consequences on the infected dog’s health. According to Nolan and colleagues, treatment with macrocyclic lactones can have a safe and off-label effect also on *D. repens* adults (69), which would explain the death of the nematode prior the surgery in this particular case. Macrocyclic lactones also affect *D. repens* mfs, as demonstrated in previous studies (67, 70). Although milbemycin oxime acts against *D. repens* microfilariae and prevents any further *D. repens* infection (71, 72), its adulticidal activity has not yet been confirmed. Nonetheless, in a previous study conducted by Giudice and colleagues, a correlation was noted between the inflammatory response of a dog infected with multiple subcutaneous dirofilariosis nodules and milbemycin oxime administration, which presumably prevented the nematodes from further development (71). Additional investigations are needed to fully understand this process and its impact on management of this VBP.

According to the European Scientific Counsel on Companion Animal Parasites (ESCCAP) guidelines, before travelling from endemic to non-endemic areas, dogs should be examined and, if the infection is detected, treated for both dirofilarial infections. Conversely, for travel from non-endemic to endemic areas, it is recommended to start a monthly preventive treatment of dogs and cats with macrocyclic lactones 30 days prior to entering the risk area. For long stays in endemic areas (i.e., more than 1 month), the administration should occur every month or every 12-weeks for cats with an extended duration spot-on macrocyclic lactone, with the last dose given after return to a non-endemic country (73). Furthermore, animals with unknown history should receive prophylactic treatment for 2 months in order to kill any migrating L3 and L4 and should be retested after 6 and 12 months after arrival in the new country (73, 74). As reported by the Animal and Plant Health Agency (APHA), more than 66,000 dogs were commercially imported into the United Kingdom in 2020, with a concomitant rise in ‘low-welfare’ importation practices and smuggling activities (61). Rising imports and falling standards of husbandry can only serve to increase the prevalence of exotic parasite infections in dogs resident in the United Kingdom. As for the *Dirofilaria* case reported by Agapito and colleagues, this nematode was initially misdiagnosed as *T. callipaeda* in an aberrant location (59). Interestingly, the two adult nematodes species are very different in their morphometric characteristics and in the clinical signs they cause (75). Similar to *Dirofilaria*, *T. callipaeda* is a European VBP which is undergoing a shift in its epidemiology (76). Only imported cases of thelaziosis have been recorded to date in the United Kingdom, but there appears to be a better awareness of this parasite, its life cycle and its distribution among United Kingdom veterinarians compared to *Dirofilaria* spp. Thus, there exists a clear need to reinforce awareness of dirofilariasis in the United Kingdom in both veterinary and medical fields. This should be accompanied by the ongoing publication of bulletins reporting parasite and disease distribution together with the creation of ‘easy to access’ resources documenting new clinical cases of exotic VBP disease together with guidelines on the diagnostic approaches which should be employed. The risk of the establishment of hitherto exotic filarial nematodes in the United Kingdom is a realistic and ongoing concern. New dirofilarial ‘hot spots’ have emerged in central Europe in recent years (2, 4, 13) and environmental conditions and vector availability appear more permissive for *Dirofilaria* spp. to encroach into northern European countries than in the past (11, 42, 77). In fact, as reported by Medlock and colleagues, modelling studies have predicted that certain areas as the United Kingdom are becoming sufficiently warm for the survival of invasive mosquitoes such as *Aedes albopictus*, known to be a competent vector of *Dirofilaria* spp. (42, 78). Therefore, in 2010 the Health Protection Agency (HPA) and colleagues started an intensive surveillance programme to investigate the presence of invasive mosquito species in England, Wales, Scotland and Northern Ireland (42). *Ae. albopictus* eggs were detected in the United Kingdom for the first time in 2016 and then again in 2017 and 2018, although no *Ae. albopictus* adult mosquitoes have been captured to date (77). However, *Ae. albopictus* is not the only potential vector of *Dirofilaria* spp.; several other *Aedes* and *Anopheles* mosquitoes have established in the United Kingdom which may transmit the infection when environmental conditions allow and these include *Aedes vexans*, *Aedes cinereus*, *Ochlerotatus detritus*, *Ochlerotatus caspius*, *Ochlerotatus punctor*, *Ochlerotatus sticticus*, *Finlaya geniculatus*, *Anopheles atroparvus*, *Anopheles claviger* and *An. plumbeus* (79). Other potential vectors such as *Culex pipiens* and *Culiseta annulata* have also been recently identified in this country (13, 42).

It is difficult to contend with the ecological changes extending vector and pathogen distributions and these will ultimately determine whether *Dirofilaria* spp. can definitely establish in the United Kingdom. However, it is prescient for veterinarians, physicians and pet owners to be more aware of these exotic parasites, so that they may be properly considered in the course of differential diagnosis in suspected human and animal cases.

## Author contributions

RP and WW contributed to conception and design of the study and wrote sections of the manuscript. RM referred the clinical case. RP, EM, AP, and MM performed the laboratory analyses. RP wrote the first draft of the manuscript. All authors contributed to the article and approved the submitted version.

## Conflict of interest

RM was employed by Dundas Veterinary Group Limited.

The remaining authors declare that the research was conducted in the absence of any commercial or financial relationships that could be construed as a potential conflict of interest.

## Publisher’s note

All claims expressed in this article are solely those of the authors and do not necessarily represent those of their affiliated organizations, or those of the publisher, the editors and the reviewers. Any product that may be evaluated in this article, or claim that may be made by its manufacturer, is not guaranteed or endorsed by the publisher.

## Footnotes

^1^ http://blast.ncbi.nlm.nih.gov/Blast.cgi
